# Set-base dynamical parameter estimation and model invalidation for biochemical reaction networks

**DOI:** 10.1186/1752-0509-4-69

**Published:** 2010-05-25

**Authors:** Philipp Rumschinski, Steffen Borchers, Sandro Bosio, Robert Weismantel, Rolf Findeisen

**Affiliations:** 1 Institute for Automation Engineering, Otto-von-Guericke-Universitisät Magdeburg, Magdeburg, Germany; 2 Institute for Mathematical Optimization, Otto-von-Guericke-Universität Magdeburg, Magdeburg, Germany; 3 Magdeburg Centre for Systems Biology (MaCS), Otto-von-Guericke-Universität Magdeburg, Magdeburg, Germany; 4 International Max Planck Research School (IMPRS), Max Planck Institute for Dynamics of Complex Technical Systems, Magdeburg, Germany

## Abstract

**Background:**

Mathematical modeling and analysis have become, for the study of biological and cellular processes, an important complement to experimental research. However, the structural and quantitative knowledge available for such processes is frequently limited, and measurements are often subject to inherent and possibly large uncertainties. This results in competing model hypotheses, whose kinetic parameters may not be experimentally determinable. Discriminating among these alternatives and estimating their kinetic parameters is crucial to improve the understanding of the considered process, and to benefit from the analytical tools at hand.

**Results:**

In this work we present a set-based framework that allows to discriminate between competing model hypotheses and to provide guaranteed outer estimates on the model parameters that are consistent with the (possibly sparse and uncertain) experimental measurements. This is obtained by means of exact proofs of model invalidity that exploit the polynomial/rational structure of biochemical reaction networks, and by making use of an efficient strategy to balance solution accuracy and computational effort.

**Conclusions:**

The practicability of our approach is illustrated with two case studies. The first study shows that our approach allows to conclusively rule out wrong model hypotheses. The second study focuses on parameter estimation, and shows that the proposed method allows to evaluate the global influence of measurement sparsity, uncertainty, and prior knowledge on the parameter estimates. This can help in designing further experiments leading to improved parameter estimates.

## Background

Mathematical modeling has become an important tool for analysis and prediction of metabolic and signal transduction processes [1,2]. Given a biological system and some experimental evidence, deriving a model hypothesis that captures the essential behavior of the system under study is a nontrivial task. Limited prior knowledge on the involved reaction mechanisms and signaling pathways may lead to competing structural hypotheses, whose parameters might be completely or largely unknown. Moreover, the model dynamics are typically strongly influenced by the model parameters [3,4]. An accurate parameter estimation is thus a crucial step to conclusively discriminate between structural alternatives, allowing to discard models for which it can be proved that no parametrization is consistent with the experimental evidence.

Model invalidation and parameter estimation are considerably more challenging in biology than in other experimental and engineering sciences, requiring specifically tailored methods. Experiments are usually time intensive, expensive, and very sensitive with respect to the environmental conditions and the used stimuli. As a result, typically only sparse experimental data is available, in which uncertainty may arise not only from technical measurement limitations, but also from intrinsic and essential features of the involved cellular processes, as e.g. cell variability [5], cell history [6] or limited excitability [7]. Moreover, in many cases the kinetic parameters cannot be directly determined from experiments [8].

Parameter estimation and model invalidation are often stated as optimization problems, in which some objective (or cost) function is minimized over appropriate optimization variables (e.g. the model parameters). A common objective is the minimization of the difference between measurement data and model prediction, evaluated by least squares or maximum likelihood functions (see e.g. [9]). Due to the nonlinearities typically arising in models of biological systems, the resulting optimization problems are frequently non-convex and very hard to solve. As a consequence, common approaches (see e.g. [10]) aim at finding *locally optimal *solutions, instead of *globally optimal *ones. As the local optimum found strongly depends on some initial guess, such approaches are often combined with stochastic strategies to achieve some desired global property [11-13]. Examples are evolutionary algorithms [14], multiple-shooting [15], clustering [16], and simulated annealing methods [17]. However, finite-time convergence to a global optimum is typically not guaranteed (see e.g. [18]), and within a fixed time limit one might find only unsatisfying estimates, by which the model alternatives cannot be discriminated, or no estimate at all. Interval analysis and inversion-based estimation methods (see e.g. [19-21]) can overcome some of these limitations, and handle model nonlinearities as encountered in biological systems. However, unless certain monotonicity conditions are satisfied, the results obtained are often very conservative (*wrapping effect*), or the computational costs too high. A rather novel approach proposed for model invalidation is the use of barrier certificates [22,23]. Barrier certificates are functions of state, parameters and time that separate possible model trajectories from measurement data, thus allowing to conclusively invalidate a model. However, finding a barrier certificate is a nontrivial task, and its existence is not guaranteed in general. In summary, even if significant progress has been achieved over the past decades (see also [24]), parameter estimation and model invalidation remain challenging problems, especially in the scope of systems biology. In this paper we propose a set-based framework for parameter estimation and model invalidation. Instead of searching for an optimal parameterization, we aim at directly classifying the parameter space into regions that are consistent with the measurements and regions that are not. A complete investigation of the parameter space provides a valuable complement to statistical informations. It not only allows to invalidate a model, in case no feasible parameterization is found, but can be useful, for example, to identify knockout targets, or for experimental design.

Our framework originates from a parameter estimation approach presented in [25], that considers biochemical reaction networks in which some steady state (equilibrium) has been reached. As stationary data is in general not sufficient to invalidate models or to estimate parameters (see e.g. [26,27]), we extend this technique to consider the observed transient. Furthermore, we take into account that not all concentrations are necessarily available by measurements, as it is frequently the case for the transient phase of biological experiments. The resulting approach, which can be applied to a quite general class of nonlinear dynamical systems, allows to take uncertain measurements into account, and can provide *conclusive *proofs of model invalidation. This is achieved by reformulating the model invalidation and parameter estimation tasks in terms of a nonlinear *feasibility problem*. Coupled with the use of a special class of *infeasibility certificates *obtained by semidefinite programming [28,29], and with an effective exploration strategy, this allows to efficiently outer-bound the set of consistent parameters. To balance estimate quality and computational effort, we also discuss an additional technique that improves the efficiency of our approach by dividing the overall problem in smaller subproblems.

## Methods

In this section we first review the most common modeling approach for biochemical reaction networks, resulting in nonlinear ordinary differential equation systems. For this system class, we show how to formulate the model invalidation and parameter estimation tasks in terms of a feasibility problem, taking uncertain and incomplete measurements into account. An efficient solution approach for this feasibility problem is then discussed, and embedded into a bisection algorithm whose goal is the classification of the parameter space into regions that are consistent with the (uncertain) measurements and regions that are not.

### Biochemical Reaction Network Models

Signal transduction and metabolic networks, as well as genetic processes, are often described in form of biochemical reaction networks [3]. A biochemical reaction network consists of a collection of reactions involving a given set of compounds (as e.g. substrates and products, though this distinction is somewhat artificial). As many reactions are reversible, we consider reactions in the general form

where *p*^+ ^and *p*^- ^denote the forward and the reverse reaction rate respectively, and *α*_1 _...  and *β*_1 _...  define the stoichiometric relations of the participating compounds *X*_1 _... . This general scheme holds for most metabolic networks and signal transduction processes, as by combining such reactions one can obtain arbitrarily connected networks.

If the compounds quickly distribute by diffusion in the volume under study, thus resulting in uniform concentrations, spatial and stochastic effects can be neglected. In this case, the dynamics of the reaction network can be modeled by describing the vector *ν*(*t*) of reaction fluxes (rates) as a nonlinear function(1)

depending on a *state vector *, on the reaction parameters , and on some input signals . For the case of biochemical reaction networks the state vector *x*(*t*) is the vector of concentrations of the compounds, and input signals *u*(*t*) allow to model environmental changes (as e.g. ligand concentrations, external stimuli triggering a signaling cascade, or external metabolites). Note that in some cases, e.g. for a compound whose concentration is imposed from the outside, it could also be convenient to model an input as an additional state.

For a large class of biochemical reactions, comprising both Michaelis-Menten and Hill kinetics, using the generalized mass-action rate law [30] each reaction flux *ν*_*j*_(*x*(*t*), *p, u*(*t*)) can be written as

The terms *F*_*j*_(*x, p, u*) are positive rational functions that can be used to describe enzyme-catalyzed reactions, in which for example only the concentrations of non-enzymatic substrates and products occur. These terms allow to account for various phenomena, as e.g. saturation, cooperativity, or hysteresis, that cannot be directly described by the standard mass-action kinetics obtained by setting *F*_*j*_(*x, p, u*) = 1. The temporal evolution of the compounds, if diffusion and convection is neglected, can then be described by the balance equation(2)

where *N *denotes the stoichiometric matrix constructed from the pre-factors *α*_*i *_and *β*_*j *_(see e.g. [31]). An important but often neglected fact is that, depending on the technique employed, a measurement could provide not a direct information on the value of single state components (concentrations), but rather some arbitrary aggregate information. We will therefore distinguish between the system state *x*(*t*) and the system output , which for sake of generality is defined as(3)

Two examples of biochemical reaction networks are described in the Results and Discussion Section.

### Time Discretization

Our approach is based on a reformulation of the parameter estimation and model invalidation tasks as a feasibility problem in discrete-time. This allows to avoid deriving the exact solution of the differential equations. A preliminary step then consists in approximating the model dynamics as a difference equation system, e.g. by standard numerical integration methods as Euler or Runge-Kutta discretizations. Selecting an appropriate discretization scheme is in general nontrivial, in particular for systems admitting different time scales (stiff systems), and requires a rigorous treatment of numerical stability that is out of the scope of this paper (see e.g. [32] for a numerical study on dynamical systems). Note that the discretization error introduced can be partly compensated for by adding uncertainty in the data.

We assume therefore that an appropriate discretization scheme has been decided, and in the remainder we consider integer-valued *time indexes*, rather than the real-valued time points to which they correspond.

Assuming rational reaction fluxes, the discretization of the model dynamics (2) yields a difference equation system that can be expressed as a system of polynomial implicit difference equations(4)

where  and  denote respectively the state vector (concentrations) and the input signals (stimuli) at the time index *k *∈ ℕ, while  is the parameter vector as before. The above form assumes a discretization scheme with a constant time-step. If a variable time-step discretization is used, one simply has to consider a system of difference equations *G*_*k*_(*x*_*k*+1_, *x*_*k*_, *p, u*_*k*_) = 0 depending on the time index. Note that the use of a variable time-step can allow in principle to overcome some numerical problems.

We assume that the system output  at the time index *k *∈ ℕ satisfies a similar implicit polynomial equation in the form(5)

and consider experimental measurements of this ideal output *y*_*k *_as subject to uncertainty, as this is typically the case in biological experiments. Note that discrete-time models, as e.g. population models [33], can be easily formulated into the implicit form (4)-(5).

### Model Invalidation and Parameter Estimation Approach

Let us consider an experiment, performed on the biological process under study, for which a collection  of measurements taken at the time indexes *k*_1 _<*k*_2 _< ... <*k*_*m *_(not necessarily consecutive) is available, and let  denote the collection of the corresponding applied inputs. Given a candidate model (4)-(5), we can define the following problems.

**Model Invalidation**. *Show that there exists no parameter vector for which the model is consistent with the experimental data *.

**Parameter Estimation**. *Find the set of all parameters (if any) for which the model is consistent with the experimental data *.

**Figure 1:**
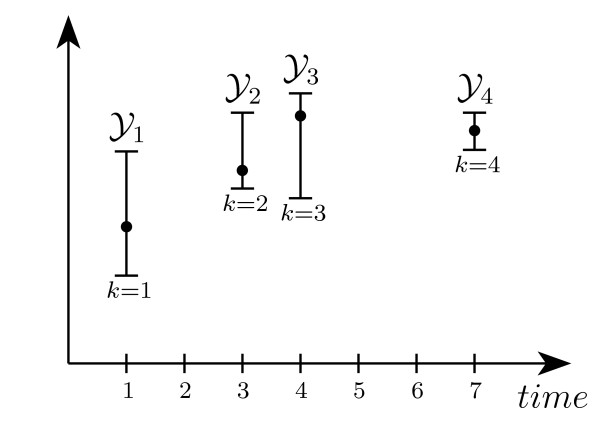
**Set-bounded measurements**. A collection of measurements  taken at different times, where the points indicate the real (unknown) measurement output *yk*, and the intervals indicate the actual measurement boundaries.

Measurements, e.g. western blots, are typically uncertain and subject to noise, so that the exact value of the output *y*_*k *_is not known. In comparison to stochastic approaches, where a measurement is seen as a probability distribution, we simply assume that each measurement  is given as a set in which the unknown output *y*_*k *_is contained (as depicted in Figure 1). Indeed, measurements are frequently given as intervals  with upper and lower bounds  and . For sake of generality, in the remainder a measurement  will be considered as an arbitrary (polyhedral) set.

Let the measurement collection be formally denoted as

where *M *= {*k*_1_,..., *k*_*m*_} is the set of the measurement index times. Assuming without loss of generality that the experiment starts with the first measurement and ends with the last one, the measurement collection  implicitly defines the *window * of time indexes for which the discrete-time dynamics (4) have to be considered. As for the measurements, the applied inputs *u*_*k *_could be subject to uncertainties, and are thus given as a collection of sets

For sake of simplicity, we assume that an input is applied at every time index *k *∈ *T*. The extension to the case in which inputs are only applied at some specific time indexes is straightforward.

Given these definitions, the model invalidation and parameter estimation problems can be more formally formulated as follows.

**Model Invalidation**. *Show that there exists no parameter **for which the conditions *(4)-(5), , and *are satisfied for all k *∈ *T*.

**Parameter Estimation**. *Find the set **of all parameters for which the conditions *(4)-(5), , *and **are satisfied for all k *∈ *T*.

### Feasibility Problem Formulation

In this section we show how to formulate and handle the model invalidation and parameter estimation problems for biochemical reaction networks in a unified framework. Assume the experimental data ,  and a candidate model (4)-(5) to be given. We can gather all this information within the following set of (semi-)algebraic equations

where  and  denote some given convex sets bounding respectively the parameters and the concentrations. Such bounds can often be derived as intervals by physical conservation relations (if the initial concentrations are known), but arbitrary regions can be assumed if only limited prior knowledge is available. The goal of parameter estimation is to provide a better approximation of the consistent parameters than these initial parameter bounds.

Checking if  admits a solution or not, which we denote as the *feasibility problem*, is equivalent to checking whether the model is able to reproduce the measurements for the given parameter set . We then clearly have the following implication:

**Property**. *If the feasibility problem **does not admit a solution, then there is no parameter vector  for which the (discrete-time) model is consistent with the experimental data , *.

Moreover, it is easy to see that the set of consistent parameters  is the set of all parameters for which the feasibility problem admits a solution (see [34] for a formal definition of the set of consistent parameters in terms of orthogonal projections). Note that the set  is not necessarily convex, and may be composed of disconnected regions.

Due to the nonlinearities of the model (4)-(5), providing an exact solution to the feasibility problem  is in general extremely hard. However, as shown in the next section, it is possible to efficiently address a *relaxed *version of the feasibility problem, where by relaxed we mean that no feasible parameterization will be lost (no false negative), although some infeasible parameterizations could be erroneously regarded as feasible (false positives). This means that there could be cases in which solving problem  would allow to invalidate a model, while solving the relaxed version does not. However, if the relaxed version is infeasible then we have the guarantee that problem  is infeasible as well, and hence that the model is inconsistent with the experimental data.

### Problem Relaxation and Infeasibility Certificates

As mentioned above, the feasibility problem  is in general a hard non-convex problem. A more tractable problem is obtained by relaxing the polynomial problem  into a semidefinite program (SDP). This approach derives from a relaxation technique proposed in [25], based on an image convexification described in [28,35]. The technical derivation of this approach, which consists of reformulating  as a quadratic problem and relaxing it into a semidefinite program, is described in detail in the Additional file 1. A comprehensive example illustrating its application is given in the Additional file 2.

The key advantage of this approach is that semidefinite programming can be solved efficiently (i.e., in polynomial time in the input size). The computational effort required in practice might pose a limit to the size of problems that can be considered. However, we are not interested in optimizing some objective function over the solutions of , but rather in deciding whether a solution exists or not. A more efficient approach can then be obtained by solving the *Lagrangean dual *of the semidefinite relaxation of , which we denote by , by standard primal-dual interior-point methods [36]. The Lagrangean *weak-duality *property guarantees that if  is unbounded then  is infeasible, thus providing an efficient certificate to model inconsistency:

**Property**. *If the Lagrangean dual **is unbounded, then there is no parameter vector  for which the (discrete-time) model is consistent with the experimental data *, .

### Exploration Strategy for Parameter Estimation

If  is bounded, then there might be parameters in  that are consistent with the measurements. The goal then becomes to approximate the subset  of consistent parameters as best as possible. If for a given subregion  the Lagrangean dual  is unbounded, then the subregion  does not contain any consistent parameterization and can be safely discarded. One can then approximate  by systematically exploring subregions , removing those that are inconsistent with the measurements.

Formally, we aim at deriving the set

Note that all consistent parameters are clearly contained in .

**Figure 2:**
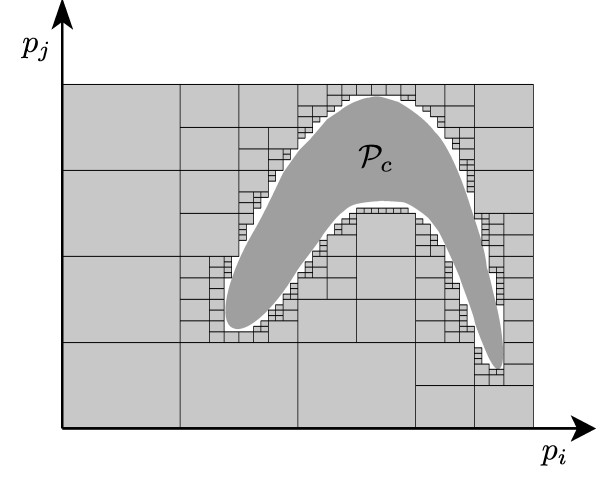
**Bisection algorithm and consistent parameter region **. The figure shows the set  and its approximation. Grey boxes indicate inconsistent parameter regions. The white set depicts the approximation of the consistent parameters.

Deriving  exactly would require to consider infinite subregions . However, restricting to any finite collection of subregions yields a valid outer-approximation of , and hence of . A simple approach to derive such an approximation is to *partition *the parameter space  and to check each partition. A more efficient approach consists in embedding the inconsistency tests within a bisection algorithm, so as to check whole groups of partitions simultaneously, as illustrated in Figure 2. Consider a given initial parameter region  and a threshold *ε *for the relative precision of the parameter estimate, and let ||*Q*|| denote the relative size of a subset  with respect to . The following simple bisection algorithm explores the parameter space in a robust and convergent manner:

**Algorithm 1: Outer-Approximate **

1. If  is unbounded then exit and return 

2. If  then exit and return 

3. Partition  into  and 

4. Set : = Outer-Approximate 

5. Set : = Outer-Approximate 

6. Return 

The overall computational cost grows exponentially in the dimension of  as well as on the threshold ε. On the other side, the algorithm can be easily and efficiently parallelized. Let us remark that a simple bounding-box approximation of the consistent parameters, which in some cases might be sufficient, can be obtained in polynomial time by separately estimating each single parameter.

The complexity of the proposed method also depends on the number of measurements considered and on the size of the corresponding time window. This is exploited in the reduction strategy described in the next section, which allows to tackle larger problems.

### Complexity Reduction for a Large Number of Measurement Points

**Figure 3:**
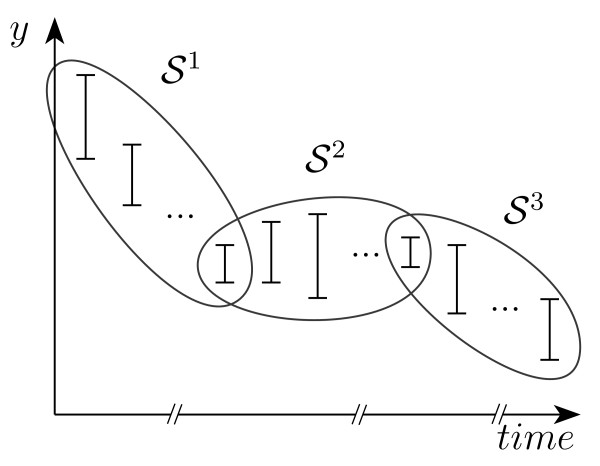
**Measurement subsequences**. To reduce the computational costs, the measurements can be split into smaller subsequences.

The key idea, as depicted in Figure 3, is to split the measurement sequence  into a collection

of shorter measurement subsequences, possibly overlapping. Each subsequence  identifies a smaller time window , and the corresponding feasibility problem  is a restricted version of  with only variables and constraints for *k *∈ *T*^*j *^, which is smaller and thus easier to solve.

**Figure 4:**
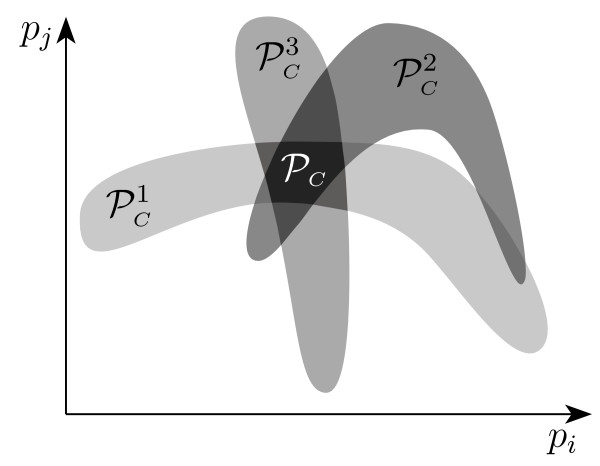
**Measurement subsequences: consistent parameters**. Consistent parameter sets for the measurement subsequences, whose intersection contains the set of consistent parameters .

It is straightforward to see that whenever *any *single problem  is infeasible, then the global problem  is infeasible as well (although the converse is not necessarily true). More in general, the set of consistent parameters  can be bounded by intersecting the subsets  of parameters that are consistent with the measurements contained in  (as depicted in Figure 4), which in turn can be approximated with the sets  derived with the algorithm described above. Namely, we have that

This strategy allows therefore to obtain an estimate on the consistent parameters even when a direct solution of the feasibility problem  is not possible, because it is computationally too expensive.

## Results and Discussion

In the previous sections we have shown how the model invalidation and parameter estimation problems can be handled in a uniform framework in terms of nonlinear feasibility problems. In this section we provide two case studies illustrating its application. In the first one we consider two simple alternative reaction schemes, namely the Michaelis-Menten and the Henri mechanisms, and aim at invalidating the first scheme with respect to uncertain measurements corresponding to the second one. In the second case study we apply the approach to an intracellular shuttling mechanism, focusing on parameter estimation under uncertain and incomplete measurements.

### Model Discrimination between Henri and Michaelis-Menten Mechanisms

Let us consider a certain enzyme-catalyzed reaction, in which an enzyme (E) and a substrate (S) join into an enzyme-substrate complex (C) to form a final product (P). Let the hypotheses proposed for this process be the two models formulated by Henri in 1902 [37], respectively known as the Michaelis-Menten (MM) and the Henri (H) mechanism of enzyme-catalyzed reaction:

where *p*_*i *_and  are the rate constants. The relevance of these two models is discussed in [26], in which it is also proved that they are analytically indistinguishable in steady state conditions, and can only be distinguished if a transient initial dynamic is considered.

The MM reaction mechanisms are modeled according to the law of mass action by

while for the H mechanism we obtain

Exploiting two conservation relations fulfilled by both mechanisms, the models can be simplified into second order systems depending only on the concentration of *S *and *C *(see the Additional file 2). Considering a simple first order Euler discretization scheme, and fixing the total enzyme concentration *E *+ *C *to a constant value 1, the difference equations corresponding to the MM mechanism are given by

where *h *is the time-step of the discretization, while for the Henri mechanism we obtain

#### Scenario and Setup

To show that our approach allows to prove model invalidity, we assume the Henri mechanism as reference, generate measurements by sampling a simulation during the transient phase, and use the resulting data for model invalidation against the Michaelis-Menten model.

The discrete-time model for the Henri mechanism has been simulated with time-step *h *= 0.1 seconds and parameters  for several initial conditions *x*_0 _= (*s*_0_, *c*_0_), deriving for each a corresponding sequence of states *x*_*k *_= (*s*_*k*_, *c*_*k*_), for *k *= 0,...,20. Given a state sequence (*x*_*k*_) and a measurement error σ, we denote by  the corresponding uncertain measurement sequence, with measurement sets . To test if the sequence  allows to invalidate the Michaelis-Menten mechanism, we apply Algorithm 1 with precision threshold *ε *= 5%, using as bounds for the unknown parameters the interval set . If the resulting parameter set is empty, the Michaelis-Menten mechanism is invalidated.

#### Results and Discussion

In Table 1 we report, for seven different initial conditions, the highest measurement error σ for which our approach allows to invalidate the Michaelis-Menten mechanism. The measurement error decreases as the initial conditions approach the steady state (recall that in the steady state the two systems are indistinguishable [26]). Comparing these results with the practical measurement errors that can be obtained in enzymological assays (see e.g. [38-40]), invalidation can be achieved when the system is sufficiently excited.

**Table 1:** Model invalidation results for the Michaelis-Menten mechanism

Initial Conditions		Maximum Error
**substrate (*s***_**0**_)	**complex (*c***_**0**_)	σ [%]
0.999	0.001	±14.0%

0.990	0.010	±13.0%

0.900	0.100	±8.5%

0.800	0.100	±8.0%

0.800	0.200	±5.0%

0.700	0.300	±2.5%

0.600	0.400	±0.5%

Results of the model invalidation of the Michaelis-Menten mechanism. For each choice of initial conditions (concentration of substrate and complex), we report the largest error σ (in percentage) for which the resulting uncertain measurements  allow to invalidate the Michaelis-Menten model.

### Parameter Estimation for a Carnitine Shuttle Mechanism

In this section we apply the proposed parameter estimation approach to the carnitine shuttle mechanism, a well known intracellular transport system for fatty acids. This example demonstrates the influence exerted by uncertainty, sparsity and incompleteness of measurements, and by prior knowledge, on the quality of the parameter estimates.

**Figure 5:**
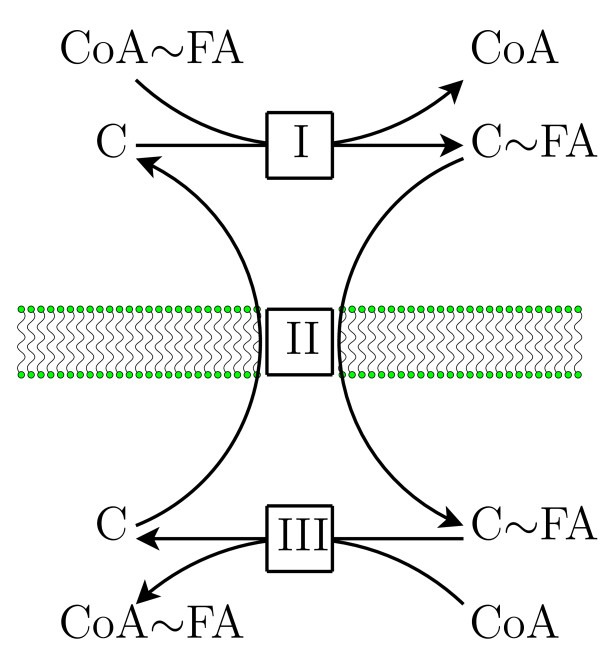
**Carnitine shuttle**. Scheme of the carnitine shuttling system. Activated fatty acid (CoA~FA) are transferred to carnitine (C) via carnitine-acyltransferase [I] at the cytoplasm. The carnitine-fatty acid complex (C~FA) is then shuttled via a so called antiporter [II] into the mitochondria in exchange for a free carnitine. There, a mitochondrial isoform of the carnitine-acyltransferase [III] reactivates via Coenzyme A (CoA) the fatty acids. The activated fatty acid inside the mitochondria is a precursor for *β*-oxidation. Note that reactions [I] and [III] are reversible.

The carnitine shuttle, as a step of mitochondrial *β*-oxidation, is an important mechanism for fat catabolism. The considered reaction scheme (see Figure 5) is adapted from [41], and models a specific transport system at the inner mitochondrial membrane involving fatty acids (FA), carnitine (C) and Coenzyme A (CoA). An activated fatty acid (CoA~FA) is transferred to carnitine (C) via carnitine-acyltransferase at the cytoplasm (reaction I). The carnitine-fatty acid complex (C~FA) is then shuttled via a so called antiporter into the mitochondria in exchange for a free carnitine (reaction II). There, a mitochondrial isoform of the carnitine-acyltransferase reactivates via Coenzyme A (CoA) the fatty acids (reaction III). The activated fatty acid inside the mitochondria is a precursor for *β*-oxidation. Note that reactions I and III are reversible.

By considering mass action kinetics and taking into account the conservation moieties [41], the dynamic of the shuttle system can be expressed by the following ordinary differential equations

where the variables *x*_1 _... *x*_4 _correspond to the participating compounds (as described in Table 2), the parameters *p*_1 _... *p*_5 _denote the (unknown) constant reaction rates, *C*_0 _and  represent the initial concentrations of carnitine respectively outside and inside the mitochondria, and the input *u *is regarded as a binary function corresponding to active (*u *= 1) and inactive (*u *= 0, fat starvation) *β*-oxidation. Applying Euler discretization, the difference equations for the above continuous-time model are given by

**Table 2:** Variables for the carnitine shuttle model

Symbol	Specie
*x*_1_	CoA~FA (Cy)

*x*_2_	Carnitine (Cy)

*x*_3_	CoA~FA (Mi)

*x*_4_	Carnitine (Mi)

Description of the variables for the carnitine shuttle model.

where *h *is the time-step, and for simplicity the time index is given in superscript.

#### Scenarios and Setup

The discrete-time model has been simulated with time step *h *= 5 seconds using the reference parameterization p* and initial condition as in Table 3, with values chosen from the literature [42,43]. To test the robustness of the approach and study the influence of measurement quality and availability on the resulting estimates, we compare several experimental scenarios derived from the above simulation. Each scenario is obtained as a combination of the following options, as summarized in Table 4.

**Table 3:** Simulation parameters and conditions for the carnitine shuttle model

Symbol	Value	Unit
	5.00e-4	*μMs*^-1^

	1.03e-1	*μ*(*Ms*)^-1^

	2.36e-2	*s*^-1^

	1.85e-2	*μ*(*Ms*)^-1^

	2.50e-2	*s*^-1^

*C*_0_	0.33	*μM*

	1.00	*μM*

Reference values for parameters and initial conditions for the simulation of the carnitine shuttle model. The initial conditions are given by *x*_1 _= 0, *x*_2 _= *C*_0_, *x*_3 _= 0 and *x*_4 _= .

**Table 4:** Carnitine shuttle example: scenarios

Scenario Type	Options			
prior knowledge	3-PAR			5-PAR

measurement density	DENSE			SPARSE

measurement error	ERR-1%		ERR-2%	ERR-4%

measured concentrations	ALL	NOT-X3	NOT-X4	NOT-X3-X4

Summary of the measurement and knowledge options. Each parameter estimate scenario is obtained by selecting a value for each of the options.

• **Prior knowledge**. Two prior knowledge options, denoted 3-PAR and 5-PAR, are considered. In the former, parameters *p*_1 _and *p*_5 _are known with relative bounds [0.95, 1.05], while parameters *p*_2_, *p*_3_, *p*_4 _are unknown. In the latter, all five parameters are unknown. For the unknown parameters we assume as initial bounds the relative bounds . *C*_0 _and  are treated in the difference equations as constants, with values as in Table 3. Here relative bounds [*lb, ub*] for a parameter *p*_*i *_mean .

• **Measurement density**. We consider two measurement density options, denoted DENSE and SPARSE. The former consists of two sequences of 15 consecutive measurements each, taken in the transient (*k *= 0,..., 14) and in the equilibrium (*k *= 300,..., 314) phase, respectively. The latter consists of two sequences of only five measurements each, taken in the transient (*k *= 0, 3, 5, 10, 14) and in the equilibrium (*k *= 300, 303, 305, 310, 314) phase, respectively.

• **Measurement errors**. To analyze the influence of measurement errors, we consider the three options ERR-1%, ERR-2%, and ERR-4%, with respectively 1%, 2% and 4% relative error (see [44,45] for examples of practical measurement errors compatible with our setup).

• **Measured concentrations**. The influence of incomplete measurements is also investigated. We consider four different options, denoted ALL, NOT-X3, NOT-X4, and NOT-X3-X4, where respectively all concentrations, all concentrations but *x*_3_, all concentrations but *x*4, and only concentrations *x*_1 _and *x*_2 _are measured. This choice reflects the fact that the inner mitochondrial concentrations *x*_3 _and *x*_4 _are more difficult to measure with simple techniques.

**Figure 6:**
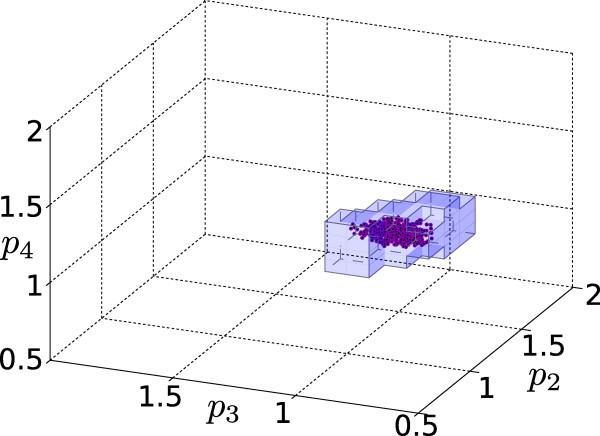
**Carnitine shuttle example: small error scenario**. Consistent parameter estimate for the scenario (3-PAR, DENSE, NOT-X3, ERR-1%). The dots show consistent Monte Carlo parameterizations. The coordinate axes show values relative to the reference parameter p*.

**Figure 7:**
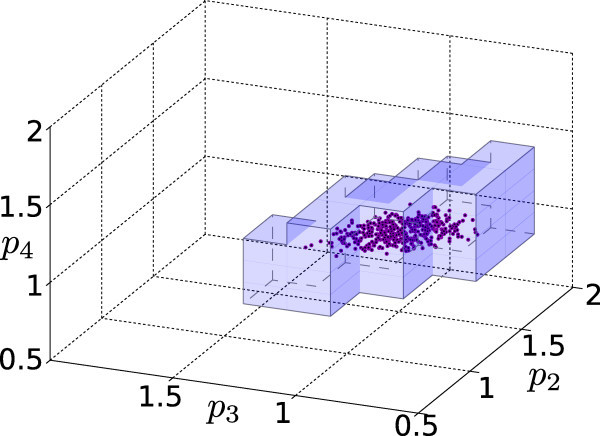
**Carnitine shuttle example: medium error scenario**. Consistent parameter estimate for the scenario (3-PAR, DENSE, NOT-X3, ERR-2%). The dots show consistent Monte Carlo parameterizations. The coordinate axes show values relative to the reference parameter p*.

For each of the resulting 2^2 ^× 3 × 4 = 48 different experimental scenarios, the consistent parameters are estimated by means of Algorithm 1, with precision threshold *ε *= 5%. The solution of each Lagrangean dual in Algorithm 1 takes in average approximately 2 minutes on a standard 2.4 GHz Intel desktop with 4 GB RAM, using a straightforward Matlab implementation (see the Additional file 2). As an example, the results in Figure 6 and 7 involved the solution of ~150 dual problems. Note that ad hoc optimizations of the semidefinite solver can strongly reduce the computing time (see e.g. [46]), as well as adaptations of Algorithm 1 to special structures, e.g. avoiding to explore the interior of large feasible regions.

#### Results and Discussion

**Figure 8:**
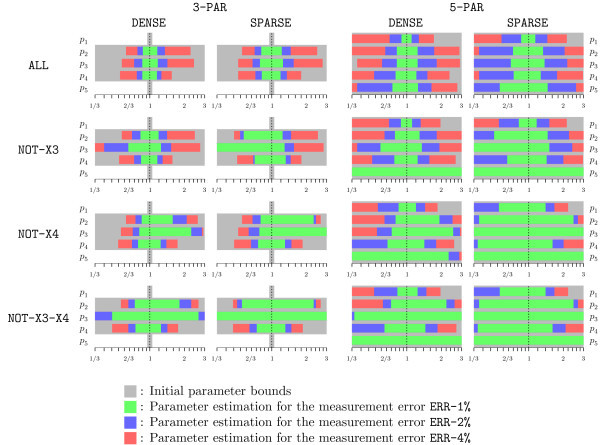
**Parameter estimation for the carnitine shuttle example**. Parameter estimation results for the carnitine shuttle example. In the first group of tests the parameters *p*_1 _and *p*_5 _are known (relative bounds fixed to [0.95, 1.05]), and the three remaining ones are unknown (initial relative bounds ). In the second group all five parameters are unknown. The rows report the parameter estimation results for different state-measurement scenarios.

The relative bounds resulting from parameter estimation are summarized in Figure 8 for all the considered scenarios. The figure is structured in a table-like fashion, with groups of experimental scenarios arranged from highest information (top-left) to lowest information (bottom-right). In each group, the bounds for the three error-measurement options are reported as nested intervals, using different colors.

The results clearly indicate that the measurement error has a substantial impact on the estimates. With measurement error ERR-1%, the unknown parameters can be narrowed with sufficient precision for most scenarios. Conversely, with measurement error ERR-4%, reasonable estimates can only be obtained for the 3-PAR case, where the additional prior knowledge compensates for the larger uncertainty.

As for the influence of incomplete measurements, while clearly the best results are obtained when all species are measured (ALL), some improvements can still be obtained with incomplete measurements, in particular for the case NOT-X3. Note however that the bounds on parameter *p*_5_ cannot be improved when *x*_3 _is not measured (cases NOT-X3 and NOT-X3-X4), as *p*_5 _only appears in the difference equation of *x*_3_. Considering the 3-PAR case, it is also interesting to note that the cases NOT-X3 and NOT-X4 have opposite effects on the estimates, improving more the upper and the lower parameter bounds respectively. As a remark, we noted in our tests that uncertainties with respect to *x*_2 _(the carnitine-FA complex) have overall the largest impact on the quality of the parameters estimates.

Comparing the SPARSE and DENSE scenarios, it can be seen that very similar results are obtained when prior knowledge is available (3-PAR). As it can be expected, the impact of measurement errors is in general more noticeable for the SPARSE cases.

The bounds in Figure 8 are the single-parameter projections of the actual bounding sets obtained with Algorithm 1. These sets, which provide additional information on the correlation among the parameters, are illustrated for the scenarios (3-PAR, DENSE, NOT-X3, ERR-1%) and (3-PAR, DENSE, NOT-X3, ERR-2%) in Figures 6 and 7 respectively. To indicate the estimate quality, some consistent parameterizations derived by Monte Carlo simulations are also plotted. Note that this is a qualitative comparison, as the probability of finding a consistent parameterization is not uniform. Conversely, our approach guarantees that outside of the depicted regions there is no consistent parameterization.

In conclusion, this case study shows how measurements quality affects the estimation results. Note that some estimates might be improved by considering the unmeasured states as additional bisection variables. This however increases the computational effort, and a trade-off has to be found. Note also that, for the 15 scenarios in which all bounds are strictly improved, the estimates in Figure 8 are guaranteed to hold also when considering larger initial bounds for the unknown parameters.

## Conclusions

We studied model invalidation and parameter estimation problems for a quite general class of biochemical reaction systems as they typically appear in systems biology, and proposed a solution approach that yields conclusive results even with uncertain measurements and model parameters. Our method allows to take uncertain but set-bounded measurable inputs and disturbances into account. The achievable results will however depend on the problem at hand. If for instance only few measurements with large uncertainty are given, a successful result will rely on the available prior knowledge. Let us remark that limited identifiability with respect to measurement and parameter uncertainties is an intrinsic limit when dealing with guaranteed bounds.

The key to our framework is the formulation of model invalidation and parameter estimation in terms of a non-convex feasibility problem. For the considered class of polynomial/rational systems, efficient infeasibility certificates are then derived by semidefinite programming relaxation. These certificates allow to *prove *model invalidity, and are used to outer-bound the consistent parameter space by means of a bisection algorithm that systematically discards (parameter) regions that are not consistent with the experimental data, while guaranteeing that *no valid solution is lost*. This property assures, in contrast to other methods, the *global *validity of our results. In contrast to [25], from which our work is inspired, we allow for dynamic measurement data, which is in general necessary for model invalidation and parameter estimation. Furthermore, we allow for an arbitrary system output to be considered.

We demonstrated our approach with two examples of biochemical processes, showing that it can perform model discrimination and provide good parameter estimates, even if only incomplete and uncertain measurements are available. The examples also show that the method allows to evaluate the influence of measurement density, uncertainty, and prior knowledge on the parameter estimates from a global perspective. Such a rigorous analysis can help in designing experiments, or in identifying which states should be measured, to obtain better estimates. Furthermore, it can be applied to parameter sensitivity analysis, as it has been done for the stationary case [47], or extended to include discrete parameters as in [48]. Experimental design and sensitivity analysis for the dynamic case will be subject of future work. Besides parameter estimation, the method can be easily modified to assess the consistent model state space, so as to estimate for instance the model states that cannot be determined experimentally. This is done by including the desired states as (additional) bisection variables (see [34,49] for further details). A major challenge that has to be considered when applying the method is computational tractability. Even with the proposed complexity reduction approach, which splits the data in smaller blocks that can be processed in parallel, the computational cost for large problems might be limiting. In practice, it could be necessary to reduce the number of bisection variables by separately exploring selections of parameters (or even single parameters), possibly improving the estimates in an iterative fashion. It is also worth pointing out that custom codes for semidefinite programming could drastically reduce the computational time, as suggested by recent results for automatic code generation [46].

## Authors' contributions

PR, SBor, and SBos designed the study and prepared the manuscript. SBor and PR implemented the approach and performed the simulation studies. RW participated in the design and in revising the draft. RF proposed the topic and basic idea, coordinated the project, and contributed in designing and revising the manuscript. PR and SBor equally contributed to the work. All authors have read and approved the final manuscript.

## Supplementary Material

Additional file 1**Semidefinite programming relaxation**. This file provides a detailed description of the relaxation procedure, explaining the steps necessary to define the Lagrangean dual  starting from the feasibility problem .Click here for file

Additional file 2 **Application example: Michaelis-Menten**. This file provides a complete description of the application of our framework to the model invalidation of the Michaelis-Menten reaction mechanism. Click here for file
