# Supplementary material for: Set-base dynamical parameter estimation and model invalidation for biochemical reaction networks
Source: BMC Syst Biol. 2010 May 25;4:69. doi: 10.1186/1752-0509-4-69 (PMC2898671; doi:10.1186/1752-0509-4-69)
Supplement: Additional file 1 — Semidefinite programming relaxation. This file provides a detailed description of the relaxation procedure, explaining the steps necessary to define the Lagrangean dual starting from the feasibility problem . [file 1752-0509-4-69-S1.PDF]

Semidefinite Program Relaxation

Given the feasibility problem $F(\mathcal{P})$, the first step is to reformulate the polynomial feasibility problem as a quadratic feasibility problem (QFP). To do so, we construct a hierarchical decomposition of the monomials appearing in H and G in terms of products of other monomials. Formally speaking, this decomposition is defined by a set $\mathcal{B}$ of monomials in p, x, y and w satisfying the following conditions:

1. for every monomial q appearing in $F(\mathcal{P})$ there exist $r_1, r_2 \in \mathcal{B}$ such that $q = r_1 \cdot r_2$.
2. for every $q \in \mathcal{B}$ having degree at least 2, there exist $r_1, r_2 \in \mathcal{B}$ with lower degree such that $q = r_1 \cdot r_2$;
3. $1 \in \mathcal{B}$ (i.e., the constant monomial 1 is in the set);

We can then define a variable vector ξ representing the monomials in $\mathcal{B}$, where the first component ξ_1 corresponds to the constant monomial 1. Note that there are several degrees of freedom in building such a decomposition.

Let $n_\xi = |\mathcal{B}|$, and let S^{n_ξ} be the set of real symmetric matrices $n_\xi \times n_\xi$, with $\succeq$ denoting the order operator with respect to the cone of positive semidefinite matrices in S^{n_ξ}. Then, the equality constraints

$$G(x_{k+1}, x_k, p, w_k)_j = 0 \qquad k \in T_b, j \in N_g = \{1, \dots, n_g\}$$
$$H(y_k, x_k, p, w_k)_j = 0 \qquad k \in M, j \in N_h = \{1, \dots, n_h\},$$

appearing in $F(\mathcal{P})$, where n_g and n_h are the number of equations in G and H, can be written as

$$\xi^T Q_k^j \xi = 0 \qquad k \in T_b, j \in N_g$$
$$\xi^T R_k^j \xi = 0 \qquad k \in M, j \in N_h,$$

for appropriate symmetric matrices $Q_k^j, R_k^j \in S^{n_\xi}$.

The bounds $p \in \mathcal{P}$, $x_k \in \mathcal{X}$, $w_k \in \mathcal{W}_k$ and $y_k \in \mathcal{Y}_k$ appearing in $F(\mathcal{P})$ can be easily expressed by means of a set of linear constraints $A\xi \geq 0$, for a suitable matrix A. In practice, it turns out to be useful to provide the explicit upper and lower bounds for all the components of ξ, so that $A \in \mathbb{R}^{2n_\xi \times n_\xi}$. Finally, in the quadratic decomposition defined by $\mathcal{B}$, some monomials are defined as products of lower degrees monomials. Denoting by n_d the number of such dependencies, we can express them in the quadratic form

$$\xi^T D_j \xi = 0 \qquad j \in N_d = \{1, \dots, n_d\}.$$

The feasibility problem $F(\mathcal{P})$ can then be reformulated as

$$QFP(\mathcal{P}): \begin{cases} \text{find } \xi \in \mathbb{R}^{n_\xi} \text{ s.t.} & \\ \xi^T Q_k^j \xi = 0 & k \in T_b, j \in N_g \\ \xi^T R_k^j \xi = 0 & k \in M, j \in N_h \\ \xi^T D_j \xi = 0 & j \in N_d \\ \xi_1 = 1 & \\ A\xi \geq 0, & \end{cases}$$

Note that this quadratic decomposition is always possible for the considered polynomial structure.

Problem $QFP(\mathcal{P})$ can then be subsequently relaxed into a convex semidefinite program (SDP, see e.g. [29]) by setting $X = \xi \cdot \xi^T$ and replacing the conditions $\text{rank}(X) = 1$ and $\text{tr}(X) \geq 1$ with the weaker constraint $X \succeq 0$, resulting in the relaxed formulation

$$SDP(\mathcal{P}): \begin{cases} \text{find } \ X \in S^{n_\xi} \text{ s.t.} & \\ \text{tr}(Q_k^j X) = 0 & k \in T_b, j \in N_g \\ \text{tr}(R_k^j X) = 0 & k \in M, j \in N_h \\ \text{tr}(D_j X) = 0 & j \in N_d \\ \text{tr}(e_1 e_1^T X) = 1 & \\ AXe_1 \geq 0 & \\ AXA^T \geq 0 & \\ X \succeq 0, & \end{cases}$$

where $e_1 = (1, 0, \ldots, 0)^T \in \mathbb{R}^{n_\xi}$. As the relaxation process is conservative, each feasible solution for $F(\mathcal{P}, \mathcal{X})$ corresponds to a feasible solutions for $SDP(\mathcal{P}, \mathcal{X})$. However, "false" solutions may be introduced. Although this does not lead to wrong invalidation results, it may lead to consider an invalid model as valid. Constraints $AXA^T \geq 0$ strengthen the relaxation and reduce this problem. These and other additional strengthening constraints are described in [28].

The corresponding Lagrangian dual $L_D(\mathcal{P})$ to $SDP(\mathcal{P})$ is given by

$$L_D(\mathcal{P}): \begin{cases} \max\ \omega \text{ s.t.} \\ \sum\limits_{k \in T_b} \sum\limits_{j \in N_g} \nu_k^j Q_k^j + \sum\limits_{k \in M} \sum\limits_{j \in N_h} \mu_k^j R_k^j + \\ + \sum\limits_{j \in N_d} \phi_j D_j + \omega e_1 e_1^T + e_1 \lambda_1^T A + \\ + A^T \lambda_1 e_1^T + A^T \lambda_2 A + \lambda_3 = 0 \\ \\ \lambda_1 \geq 0,\ \lambda_2 \geq 0,\ \lambda_3 \succeq 0, \end{cases} \tag{8}$$

where ν_k^j, μ_k^j, ϕ_j, ω are the Lagrangian multipliers corresponding to the equality constraints in $SDP(\mathcal{P})$, and $\lambda_1, \lambda_2 \in \mathbb{R}^{2n_\xi}$, $\lambda_3 \in \mathbb{S}^{n_\xi}$ those corresponding to the remaining constraints.

The weak Lagrangian duality property of SDPs ensures that if $L_D(\mathcal{P})$ is unbounded, then the primal problem $SDP(\mathcal{P})$ is infeasible, and hence, as the relaxation process is conservative, also that $F(\mathcal{P})$ is infeasible. Checking for unboundedness of $L_D(\mathcal{P})$ can be done efficiently with standard SDP solvers such as SeDuMi [45], if the dimension of the problem is reasonable. Note that it can also be proved that the Lagrangean dual is unbounded if and only if it has a feasible solution with $\omega > 0$, which could help in reducing the computational effort (see [42]).
