# Supplementary material for: Set-base dynamical parameter estimation and model invalidation for biochemical reaction networks
Source: BMC Syst Biol. 2010 May 25;4:69. doi: 10.1186/1752-0509-4-69 (PMC2898671; doi:10.1186/1752-0509-4-69)
Supplement: Additional file 2 — Application example: Michaelis-Menten. This file provides a complete description of the application of our framework to the model invalidation of the Michaelis-Menten reaction mechanism. [file 1752-0509-4-69-S2.PDF]

Application Example: Michaelis-Menten

To give a clearer understanding of our framework, we provide all the intermediate technical steps necessary for applying the framework to the model invalidation of the Michaelis-Menten reaction mechanism

$$E + S \underset{p_2}{\overset{p_1}{\rightleftharpoons}} C \overset{p_3}{\rightarrow} E + P.$$

For simplicity of the notation we define the state vector x to be composed of the concentrations

$$x^T := \left[\begin{array}{cccc} S, & C, & E, & P \end{array}\right],$$

denoting with x_i the i-th element of the vector x. The timely evolution of the concentrations is given by the following ordinary differential equations

$$\begin{aligned}
\frac{dx_1}{dt} &= -p_1 x_1 x_3 + p_2 x_2 \\
\frac{dx_2}{dt} &= +p_1 x_1 x_3 - p_2 x_2 - p_3 x_2 \\
\frac{dx_3}{dt} &= -p_1 x_1 x_3 + p_2 x_2 + p_3 x_2 \\
\frac{dx_4}{dt} &= +p_3 x_2.
\end{aligned}$$

By considering the moiety conservation equations

$$\frac{dx_2}{dt} + \frac{dx_3}{dt} = 0$$

and

$$\frac{dx_1}{dt} + \frac{dx_2}{dt} + \frac{dx_4}{dt} = 0$$

it is possible to reduce the above 4th order system and to express it as a 2nd order system. A further simplification could be to consider a quasi-steady or a quasi-equilibrium state, but as the Michaelis-Menten is indistinguishable from the Henri kinetics under such conditions we do not apply it. We also assume that both concentrations x_1, x_2 can be measured, thus $y = [x_1 \; x_2]^T$.

If we now consider a standard Euler discretization scheme with time-step h

$$\dot{x}(k) \approx \frac{x[k+1] - x[k]}{h},$$

the above-mentioned 2nd order system is reformulated into the following explicit discrete model

$$x_1^+ = x_1 + hp_1[(x_2 - 1)x_1 + K_S x_2]$$

$$x_2^+ = x_2 + hp_1[(1 - x_2)x_1 + K_M x_2],$$

where $K_S = p_1/p_2$, $K_M = (p_2 + p_3)/p_1$. To follow the notation of the main article we reformulate (9) as an implicit polynomial map $G : \mathbb{R}^2 \times \mathcal{P} \to \mathbb{R}^2$ defined by

$$G_1(x,p) = x_1^+ - x_1 - hp_1[(x_2 - 1)x_1 + K_S x_2] \quad (9)$$

$$G_2(x,p) = x_2^+ - x_2 - hp_1[(1 - x_2)x_1 + K_M x_2] \quad (10)$$

and

$$H_1(x,p) = x_1 \quad (11)$$

$$H_2(x,p) = x_2. \quad (12)$$

Note that this reformulation is possible also for rational functions, by multiplying with the right-hand side denominator. As an example, for a Michaelis-Menten equation

$$x_4^+ = x_4 + h \cdot \frac{v_{max} x_1}{K_M + x_1},$$

with v_{max} being the maximum reaction rate under the quasi-steady-state assumption and K_M the Michaelis-Menten constant, a polynomial implicit formulation could be given by

$$G(x,p) = x_4^+(K_M + x_1) - x_4(K_M + x_1) - h \cdot v_{max} \cdot x_1.$$

As a first step for relaxing the feasibility problem into a semidefinite program, one first defines a quadratic decomposition of the monomials appearing in G. Due to several degrees of freedom, such a decomposition is not unique. A possible monomial vector ξ for our example would be

$$\xi^T = \left[\ 1,\quad hp_1,\quad hp_2,\quad hp_3,\quad x_1,\quad x_2,\quad hp_1x_2,\quad x_1^+,\quad x_2^+\ \right].$$

As an example of the above-mentioned degrees of freedom, an equivalent decomposition is obtained by removing the monomial $h \cdot p_1 \cdot x_2$ and including in its place the monomials $h \cdot x_1 \cdot x_2$ and h. Note that, in principle, the monomial vector could be constructed from all monomials appearing in G and H. However, this would lead to an SDP of unnecessary large size.

Given the monomial vector ξ, the polynomial maps (9),(10) can be reformulated in the quadratic form

$$G_j = \xi^T Q_j \xi,$$

for matrices $Q_1, Q_2 \in \mathbb{R}^{9\times 9}$ defined by

$$Q_1 = \begin{bmatrix} 0 & 0 & 0 & 0 & -0.5 & 0 & 0 & 0.5 & 0 \\ 0 & 0 & 0 & 0 & -0.5 & 0 & 0 & 0 & 0 \\ 0 & 0 & 0 & 0 & 0 & -0.5 & 0 & 0 & 0 \\ 0 & 0 & 0 & 0 & 0 & 0 & 0 & 0 & 0 \\ -0.5 & -0.5 & 0 & 0 & 0 & 0 & -0.5 & 0 & 0 \\ 0 & 0 & -0.5 & 0 & 0 & 0 & 0 & 0 & 0 \\ 0 & 0 & 0 & 0 & -0.5 & 0 & 0 & 0 & 0 \\ 0.5 & 0 & 0 & 0 & 0 & 0 & 0 & 0 & 0 \\ 0 & 0 & 0 & 0 & 0 & 0 & 0 & 0 & 0 \end{bmatrix}$$

and

$$Q_2 = \begin{bmatrix} 0 & 0 & 0 & 0 & 0 & -0.5 & 0 & 0 & 0.5 \\ 0 & 0 & 0 & 0 & -0.5 & 0 & 0 & 0 & 0 \\ 0 & 0 & 0 & 0 & 0 & 0.5 & 0 & 0 & 0 \\ 0 & 0 & 0 & 0 & 0 & 0.5 & 0 & 0 & 0 \\ 0 & -0.5 & 0 & 0 & 0 & 0 & 0.5 & 0 & 0 \\ -0.5 & 0 & 0.5 & 0.5 & 0 & 0 & 0 & 0 & 0 \\ 0 & 0 & 0 & 0 & 0.5 & 0 & 0 & 0 & 0 \\ 0 & 0 & 0 & 0 & 0 & 0 & 0 & 0 & 0 \\ 0.5 & 0 & 0 & 0 & 0 & 0 & 0 & 0 & 0 \end{bmatrix}.$$

If some monomials are defined as products of lower degree monomials, as e.g. $h \cdot p_1 \cdot x_2$ then the dependencies for these monomials have to be taken into account. So for the monomial $h \cdot p_1 \cdot x_2$ one has to specify that it really is a product of the monomials $h \cdot p_1$ and x_2, which is done by means of a constraint $D\xi = 0$, for the matrix

$$D = \begin{bmatrix} 0 & 0 & 0 & 0 & 0 & 0 & 0.5 & 0 & 0 \\ 0 & 0 & 0 & 0 & 0 & -0.5 & 0 & 0 & 0 \\ 0 & 0 & 0 & 0 & 0 & 0 & 0 & 0 & 0 \\ 0 & 0 & 0 & 0 & 0 & 0 & 0 & 0 & 0 \\ 0 & 0 & 0 & 0 & 0 & 0 & 0 & 0 & 0 \\ 0 & -0.5 & 0 & 0 & 0 & 0 & 0 & 0 & 0 \\ 0.5 & 0 & 0 & 0 & 0 & 0 & 0 & 0 & 0 \\ 0 & 0 & 0 & 0 & 0 & 0 & 0 & 0 & 0 \\ 0 & 0 & 0 & 0 & 0 & 0 & 0 & 0 & 0 \end{bmatrix}.$$

The bounds on the parameters, states and inputs can be expressed by means of a set of linear constraints in the form $A\xi \geq 0$. For this example we consider only lower and upper bounds (intervals) given in Table 1, so that we have constraints of the type $\underline{x}_1 \leq x_1 \overline{x}_1 = 1$.

**Term**	**Lower Bound**	**Upper Bound**
hp_1	$h\underline{p}_1 = 0.33h$	$h\overline{p}_1 = 3h$
hp_2	$h\underline{p}_2 = 0.33h$	$h\overline{p}_2 = 3h$
hp_3	$h\underline{p}_3 = 0.33h$	$h\overline{p}_3 = 3h$
x_1	$\underline{x}_1 = 0$	$\overline{x}_1 = 1$
x_2	$\underline{x}_2 = 0$	$\overline{x}_2 = 1$

Table 1: Upper and lower bounds on the states and parameters. Note that x_i^+ has the same bounds as x_i.

Therefore, the linear constraints $A\xi \geq 0$ defining the bounds are given by the matrix

$$A = \begin{bmatrix} -h\underline{p}_1 & 1 & 0 & 0 & 0 & 0 & 0 & 0 & 0 \\ h\overline{p}_1 & -1 & 0 & 0 & 0 & 0 & 0 & 0 & 0 \\ -h\underline{p}_2 & 0 & 1 & 0 & 0 & 0 & 0 & 0 & 0 \\ h\overline{p}_2 & 0 & -1 & 0 & 0 & 0 & 0 & 0 & 0 \\ -h\underline{p}_1 & 0 & 0 & 1 & 0 & 0 & 0 & 0 & 0 \\ h\overline{p}_3 & 0 & 0 & -1 & 0 & 0 & 0 & 0 & 0 \\ -\underline{x}_1 & 0 & 0 & 0 & 1 & 0 & 0 & 0 & 0 \\ \overline{x}_1 & 0 & 0 & 0 & -1 & 0 & 0 & 0 & 0 \\ -\underline{x}_2 & 0 & 0 & 0 & 0 & 1 & 0 & 0 & 0 \\ \overline{x}_2 & 0 & 0 & 0 & 0 & -1 & 0 & 0 & 0 \\ -h\underline{p}_1\underline{x}_2 & 0 & 0 & 0 & 0 & 0 & 1 & 0 & 0 \\ h\overline{p}_1\overline{x}_2 & 0 & 0 & 0 & 0 & 0 & -1 & 0 & 0 \\ -\underline{x}_1 & 0 & 0 & 0 & 0 & 0 & 0 & 1 & 0 \\ \overline{x}_1 & 0 & 0 & 0 & 0 & 0 & 0 & -1 & 0 \\ -\underline{x}_2 & 0 & 0 & 0 & 0 & 0 & 0 & 0 & 1 \\ \overline{x}_2 & 0 & 0 & 0 & 0 & 0 & 0 & 0 & -1 \end{bmatrix}$$

In a matrix representation every row corresponds to one inequality, e.g. row 7 of A corresponds to $\underline{x}_1 \leq x_1$. Redundant constraints of the form $AXA^T \geq 0$ can also be introduced to tighten the relaxation.

As a final step we define $X = \xi \cdot \xi^T$ and replace the conditions $rank(X) = 1$ and $tr(X) \geq 1$ with the weaker constraint $X \succeq 0$. Then, to certify the infeasibility of the problem, we transform the system to its Lagrangian dual (8). To do so, we need the following additional variables

$$\lambda_1 \in \mathbb{R}^{16}, \lambda_2 \in \mathbb{R}^{16\times 16}, \lambda_3 \in \mathbb{R}^{9\times 9}, \nu \in \mathbb{R}^4.$$

Note also that for this example, the unit vector e_1^T appearing in (8) is defined by

$$e_1^T = \begin{bmatrix} 1, & 0, & 0, & 0, & 0, & 0, & 0, & 0, & 0 \end{bmatrix}.$$

An computational implementation of the Lagrangian dual can then be obtained with standard tools, as for

example by using YALMIP in Matlab, coupled with a semidefinite program solver as SEDUMI. The allowed error in the semidefinite program solver should be set to a sufficiently small value, so as to overcome numerical problems which may introduce wrong solutions.
